# Bovine Cysticercosis in Dodota, Robe and Chole Municipal Abattoirs and Community Practices and Risk Perceptions Regarding Taeniasis in Ethiopia

**DOI:** 10.1002/vms3.71317

**Published:** 2026-11-06

**Authors:** Yihenew Getahun Ambaw, Ahmed Jemalo Jarso, Adem Hiko, Ambachew Motbaynor Wubaye, Assaye Wollelie Fentie, Animaw Andargie Worku, Simegnew Adugna Kallu

**Affiliations:** ^1^ Department of Veterinary Medicine College of Agricultural Sciences Woldia University Woldia Ethiopia; ^2^ College of Veterinary Medicine Haramaya University Dire Dawa Ethiopia; ^3^ Department of Veterinary Science College of Agriculture and Environmental Science Debre Tabor University Debre Tabor Ethiopia; ^4^ Department of Veterinary Science Burie Campus Debre Markos University Burie Ethiopia

**Keywords:** bovine cysticercosis, cattle, communities practice, Ethiopia, risk perception

## Abstract

**Introduction:**

Bovine cysticercosis incurs substantial economic losses through carcass condemnation and poses public health risks. However, updated, comprehensive evidence on the prevalence of bovine cysticercosis, community practices and risk perceptions regarding the prevention and control of taeniasis in Dodota, Chole and Robe towns remains scarce.

**Methods:**

An abattoir‐based cross‐sectional survey among 510 cattle and a community‐based questionnaire survey of 422 respondents were conducted in three towns from November 2022 to April 2023. The aim was to assess the prevalence of bovine cysticercosis, community practices and risk perceptions towards bovine cysticercosis and taeniasis. A routine meat inspection was conducted, along with a questionnaire survey administered through face‐to‐face interviews to collect community risk perceptions and practices related to bovine cysticercosis.

**Results:**

The overall prevalence of bovine cysticercosis was 13.50% (95% confidence interval [CI]: 10.83–16.77). Older age (adjusted odds ratio [aOR] = 16.13, 95% CI: 5.88–44.21) and poor body condition (aOR = 0.18, 95% CI: 0.09–0.38) were significantly associated with higher odds of exposure. In total, we recovered 142 cysts, of which 28.20% were viable. Community adoption of appropriate practices and desirable risk perceptions were 23.93% (95% CI: 19.94–28.30) and 34.83% (95% CI: 30.29–39.59), respectively. Female respondents had lower odds of implementing appropriate practices (OR = 0.47, 95% CI: 0.29–0.76) and having desirable risk perceptions (OR = 0.59, 95% CI: 0.39–0.90) than males. Having an elementary education or less reduced the odds of appropriate practices by 84% (OR = 0.16, 95% CI: 0.08–0.29) and having desirable risk perception by 58% (OR = 0.42, 95% CI: 0.25–0.71) compared with participants with a diploma or higher. Additionally, rural residence (OR = 0.41, 95% CI: 0.25–0.66) and Christian (OR = 1.76, 95% CI: 1.12–2.77) were significant factors influencing the adoption of appropriate practices.

**Conclusion:**

Bovine cysticercosis remains a prevalent and neglected food‐borne zoonotic disease, and community practices and risk perceptions are low in the study areas. To enhance awareness and mitigate disease transmission, targeted educational and training interventions should be implemented within a One Health framework.

## Graphical abstract

Active post‐mortem inspections at the Dodota, Robe and Chole municipal abattoirs revealed a moderately high prevalence of Bovine *Cysticercus* in slaughtered cattle. Combined with community survey data demonstrating widespread raw beef consumption and poor disease awareness, these findings highlight an urgent need for One Health interventions to break the *Taenia saginata* transmission cycle in Central Ethiopia.

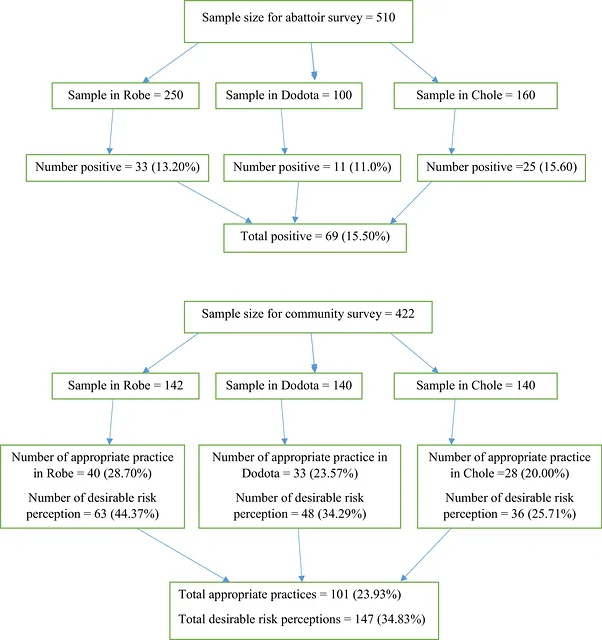

## 1 Introduction

Bovine cysticercosis is one of the most common, prevalent and neglected food‐borne zoonotic diseases in developing countries (Tsegaye et al. 2026). It causes enormous economic losses through organ condemnation and significant public health impacts. Bovine cysticercosis (beef measles) is a muscular disease of cattle caused by the larval stages of the parasite *Taenia saginata* (beef tapeworm) (Tariku and Beredo 2019). Taeniasis is a food‐transmitted disease caused by *T. saginata*, with humans as the definitive host and cattle as the intermediate host. The infection of humans by *T. saginata*, or taeniasis, occurs through the consumption of raw meat containing viable *Cysticercus bovis*. Although infection of cattle with bovine cysticercosis occurs through the consumption of feed and water contaminated with *T. saginata* eggs (Rossi et al. 2020).

The global distribution of *T. saginata* in both developed and developing countries underscores the public health importance of this parasite. However, the prevalence of the disease is more common in Africa, Latin America and some parts of Asia (Abunna 2013; MoA 2021), where the cultural practices of consuming raw meat and inadequate sanitary set‐up aggravate the likelihood of taeniasis (Fenta et al. 2025; Tsegaye et al. 2026). The epidemiology of *T. saginata* is influenced by cultural norms and ethnic backgrounds (Jorga et al. 2022), with an estimated global burden of 45–60 million cases (WHO and FAO 2014).

In Ethiopia, the excretion of faeces without proper latrines is a common practice, particularly in rural communities, where more than 80% of the population lives (MoA 2021; Jorga et al. 2022). The common traditional animal farming practices in Ethiopia primarily permit unlimited access of cattle to contaminated environments. This condition facilitates the spread of cysticercosis because cattle become infected by the ingestion of pasture/feed or water contaminated with *T. saginata* eggs (Kifle and Belete 2015). There is also evidence that the cultural habit of consuming raw meat, including *Kurt* and *Kitfo*, may contribute to the epidemiology of taeniasis in Ethiopia (Jorga et al. 2022; Tsegaye et al. 2026).

Cysticercosis in cattle is a major cause of economic losses in the beef industry due to carcass condemnation of infected meat. Among the 45–60 million cases of taeniasis worldwide, Africa accounts for approximately 40% (WHO and FAO 2014). In Ethiopia, numerous studies have reported bovine cysticercosis prevalence ranging from 3.11% to 27.6% in cattle and human taeniasis prevalence from 2.50% to 89.41% (Abunna et al. 2018; Jorga et al. 2020).

Post‐mortem examinations remain the most reliable and cost‐effective method to confirm and characterize *C. bovis* (bovine cysticercosis) (MoA 2021; Fesseha and Asefa 2023); however, they have relatively low sensitivity (10%–50%), resulting in substantial under‐detection of infected carcasses (Sithole et al. 2019). The primary method for detecting it in abattoirs involves visual inspection of incised muscles at specific predilection sites, including the masseter muscle, heart, tongue, diaphragm and oesophagus (Fesseha and Asefa 2023). The ideal prevention and control of bovine cysticercosis and taeniasis primarily depend on improving communities’ practices and risk perceptions. Regarding this issue, communities can adopt measures such as treating tapeworm infections, enhancing sanitation and cleanliness and improving cattle‐rearing techniques to reduce the faecal‐oral transmission of many infectious diseases (Bibi et al. 2023). Evidence from surveys of public practices and risk perceptions may reveal gaps in cultural norms and behavioural patterns within communities. Identifying these issues and taking action to manage or eradicate the disease is crucial (Jorga et al. 2022). In addition, assessing the current status of bovine cysticercosis in abattoirs is also essential to mitigate its public health impact (Fenta et al. 2025).

Although several researchers in Ethiopia have reported the prevalence of bovine cysticercosis across various regions over time (Jorga et al. 2022), a critical knowledge gap remains regarding multi‐centre evidence that integrates the epidemiology of the disease, including prevalence, cyst viability and organ distribution, with localized community practices and risk perceptions. Specifically, there is limited comprehensive data on these combined parameters for the towns of Dodota, Chole and Robe. The absence of such localized baseline data significantly impedes the development of targeted, culturally sensitive control strategies to mitigate this zoonotic threat and its economic implications for livestock health. Therefore, this study aims to assess community practices and risk perceptions regarding bovine cysticercosis and taeniasis prevention and control, while concurrently determining the prevalence, cyst viability and organ distribution of bovine cysticercosis in Dodota, Chole and Robe towns. Ultimately, these findings will provide a framework for designing local health promotion programmes to manage and prevent taeniasis using a One Health approach.

## 2 Materials and Methods

### 2.1 Study Area

The study was conducted in the municipal abattoirs of Dodota, Robe and Chole towns (Figure 1) in Arsi Zone, Ethiopia. Dodota is located 50 km northeast of Asella town and 125 km southeast of Addis Ababa, with a total cattle population of 92,417. Chole district lies east of Asella town, the administrative centre of Arsi Zone, and has a cattle population of 102,506. Robe district, also in Arsi Zone, has a cattle population of 215,066 (AZAO 2023). The annual slaughtering capacity of the three abattoirs is 16,200 cattle: 7938 from Robe, 5086 from Chole and 3176 from Dodota. Over the 6‐month study period, a total of 3969, 2543 and 1588 animals were slaughtered in Robe, Chole and Dodota municipal abattoirs, respectively. The municipal abattoirs chosen for this study were selected due to their high daily cattle slaughter capacity, the limited availability of reports in these areas and logistical convenience.

**FIGURE 1:**
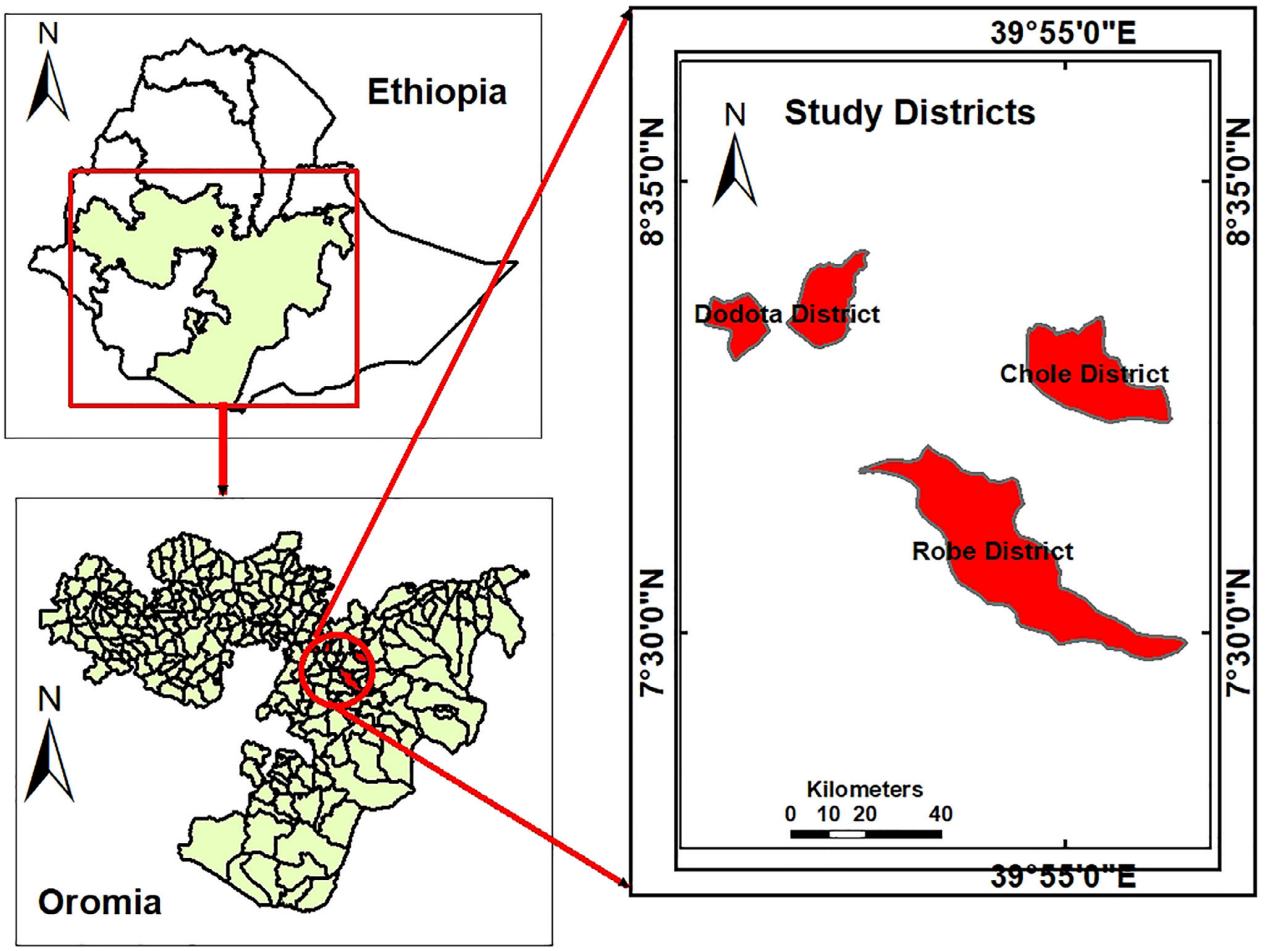
Map of the study area (Dodota, Robe and Chole districts).

### 2.2 Study Design

A cross‐sectional study was conducted from November 2022 to April 2023 to primarily estimate the prevalence of bovine cysticercosis among cattle slaughtered at municipal abattoirs in Dodota, Robe and Chole. Further information on case definition and cyst identification was found in the Supporting Information. In addition, the study also examined community practices and perceptions of risk related to bovine cysticercosis and taeniasis in the areas under investigation. The cattle brought to these abattoirs were sourced from neighbouring kebeles and districts and transported by car or on foot.

### 2.3 Sample Size Determination and Sampling Techniques

The appropriate sample size was estimated using the Thrusfield formula (Thrusfield 2018). On the basis of a previous prevalence of 37% reported at Wolayta abattoir (Regassa et al. 2009), with a 95% confidence level and 5% margin of error, the formula yielded a sample size of 358 cattle. However, to improve the reliability and applicability of the findings, the sample was inflated to 510 animals.

A systematic random sampling technique was employed to select cattle for ante‐mortem (at the lairage) and post‐mortem inspections from the three abattoirs. Allocation was proportional to the number of cattle slaughtered daily. Over the 6‐month study period, 250 cattle were sampled from Robe (out of 3969 slaughtered), 100 from Dodota (out of 1588) and 160 from Chole (out of 2543) abattoirs. Each animal was identified by labelling an ID number on the lateral aspect of its back using permanent ink. The same ID was transferred to all visceral organs after slaughter. Body condition was scored using a nine‐point method (Ndlovu et al. 2007), categorized as poor (Scores 3–4), moderate (Score 5) or good (Scores 6–9). Animals in very poor condition (Scores 1–2) were excluded as unfit for slaughter. Animal age was determined using the tooth ageing method and categorized as young (<4 years), adult (4–6 years) or old (≥7 years) (Torell 2003).

### 2.4 Sample Collection and Laboratory Analysis

Both ante‐mortem and post‐mortem inspection were performed for each selected animal at the abattoir. Disease status (abnormality) was assessed at the lairage during ante‐mortem inspection. During ante‐mortem inspection, the study animal was given a particular ID for identification purposes. After slaughter, post‐mortem inspection was carried out on each individual cattle carcass for the detection of bovine cysticercosis in accordance with the Ministry of Agriculture's recommendation (MoA 2021). This guideline comprises visualization, palpation and incision of the expected cyst predilection sites. Standardized protocols were employed during meat inspections to ensure scientific rigour and minimize bias. Researchers employed fixed anatomical checklists and uniform techniques, with training to maintain consistency in evaluations. Healthy animals were examined quickly, whereas those with abnormalities received more thorough documentation. This structured approach enhances validity and ensures uniform evaluation across all animals.

Furthermore, the inspection included a longitudinal incision of the heart from the base to the apex, two longitudinal ventral incisions of the tongue from the tip to the root, one profound incision into both sides of the triceps muscles of the shoulder, a deep incision into the external and internal muscles of the masseter parallel to the plane of the jaw, three parallel incisions into the long axes of the neck muscles on both sides and one extensive incision. The outputs of these examinations were enrolled according to each individual organ observed (MoA 2021). The detected bovine cysticercosis was incubated in ox bile at 37°C for one to 2 h with 40% ox bile solution diluted with normal saline. The scolex was investigated under a microscope for viability tests. Bovine cysticercosis was considered viable if the scolex evaginated during the incubation period; otherwise, it died (Nigatu 2004).

### 2.5 Questionnaire Design

The questionnaire was adapted from existing reports with modifications (Nyangi et al. 2022; Jorga et al. 2022; Bibi et al. 2023; Zulu et al. 2023). It consisted of 6 questions on socio‐demographic characteristics, 20 questions on practices, and 10 questions on perceptions regarding bovine cysticercosis and taeniasis. The questionnaire was designed to cover all relevant aspects of the constructs, ensuring content validity.

Internal consistency was evaluated through a pilot study with 20 respondents (later excluded from the final analysis). Cronbach's alpha values were 0.72 for the practice domain and 0.82 for the perception domain, indicating good internal consistency. Construct validity was assessed using average variance extracted (AVE), yielding scores of 0.65 and 0.59, respectively, both exceeding the 0.50 threshold for convergent validity. Factor loadings for each item exceeded to 0.40, demonstrating strong positive correlations within both domains.

The questionnaire was translated into Afaan Oromo (the respondents’ local language) and piloted among 20 respondents to assess feasibility. After adjustments, the final tool was administered to 461 respondents via face‐to‐face interviews, with 422 completing the survey (91.54% response rate).

### 2.6 Sample Size and Sampling Procedure for the Questionnaire Survey

In this survey, the minimum sample size was determined using the single proportion formula described by Thrusfield (2018). Assuming a positive practice rate of 50%, a confidence level of 95%, a margin of error of 5% and accounting for a 20% non‐response rate, the total adjusted sample size required becomes 461 respondents.

The current community‐based cross‐sectional survey was conducted in three towns. Each town is composed of kebeles, which are the smallest administrative units, further divided into villages. Villages consist of 30–50 households. We utilized a multistage random sampling method to select kebeles and villages from the three study towns. The primary sampling unit was kebeles in the town; the secondary unit was villages within the kebele, and the tertiary sampling unit consisted of households in the village. The lists of kebeles were obtained from the relevant municipalities. In Robe town, there are three urban kebeles, from which two were selected by simple random sampling using a lottery system. Chole town has four kebeles, and three were randomly chosen using the same method. Dodota town also has three kebeles, from which two were randomly selected. From the selected seven kebeles, up to three villages were randomly chosen, with the selection proportional to the size of each Kebele. In each selected village, households were selected by simple random sampling from local administrative rosters. To minimize non‐response bias, field teams made up to two follow‐up visits before replacing non‐responsive households. From the households that agreed to participate, one family member aged between 18 and 65 years was randomly selected from each household. In the lottery system, household members were allowed to draw either zero or selected, as only one individual was chosen from each household.

### 2.7 Data Measurement in the Questioners Survey

During the scoring process, all negative questions were reversed to assign the correct points. Respondents scored 1 for a correct answer and 0 for an incorrect one for both practice and risk perception questions. In the data analysis phase, a modified Bloom's cut‐off was applied. Following the tally of correct answers, respondents were divided into two groups (Subedi et al. 2023, 2025). Those who answered 75% or more correctly (15 or more questions in the practice domain and 7 or more in the risk perception domain) were classified as having appropriate practices and desirable risk perceptions. Conversely, respondents who scored below 75% (14 or less questions in the practice domain and 6 or less in the risk perception domain) were deemed to have inappropriate practices and undesirable risk perceptions (Subedi et al. 2025).

### 2.8 Data Management and Analysis

The collected raw data were cleaned, edited and verified for completeness on a daily basis after gathering. The completed raw data were recorded in Epi‐Data version 4.2, and STATA version 17 statistical software was exported for data management and analysis. The combination of descriptive (frequency and proportion) and inferential statistics (logistic regression) was employed to examine the relationships between predictor variables and the dependent variables. Univariable logistic regression was applied to compute the crude odds ratio (cOR) and to screen out candidate variables for the final model development. Each independent variable associated with bovine cysticercosis in cattle at a *p* value < 0.25 in the univariable analysis was included in the multivariable logistic regression model using a purposeful variable selection strategy (Hosmer et al. 2013) to prevent the omission of potential confounders or important covariates.

Prior to model construction, variables were evaluated for multicollinearity using the variance inflation factor (VIF), with a threshold of VIF > 7 indicating high collinearity (James et al. 2013). However, no substantial multicollinearity was observed among the candidate variables. Confounding was evaluated using the change‐in‐estimate criterion; A variable was classified as a confounder and retained if its removal induced a >10% shift in the odds ratio (OR) of primary exposure variables. Nevertheless, no confounders were identified. A similar model‐building approach was applied to analyse the community respondents’ practices and risk perceptions. Ultimately, multivariable binary logistic regression was performed to compute adjusted odds ratios (AORs) and control for potential confounding effects. Goodness‐of‐fit for the final models was assessed using the Hosmer–Lemeshow test. For all statistical analyses, a *p* value < 0.05 was considered statistically significant.

## 3 Results

### 3.1 Post‐Mortem Findings

A total of 510 cattle were selected systematically randomly to assess the prevalence of bovine cysticercosis in their internal organs. The majority of the animals in this study were from the Robe district, 250 (49.0%), followed by Chole 160 (31.4%). Most of the animals included in the study were male, totalling 396 (77.7%), and 457 (89.61%) were local breeds. The total prevalence of bovine cysticercosis was 69/510 (13.5%, 95% CI: 10.82–16.79) in cattle according to post‐mortem examination. The highest prevalence was recorded in the Chole district at 15.6% (95% CI: 10.8–22.0), whereas the lowest was observed in Dodota at 11.0% (95% CI: 6.3–18.6) (Figure 2 and Table 1).

**FIGURE 2:**
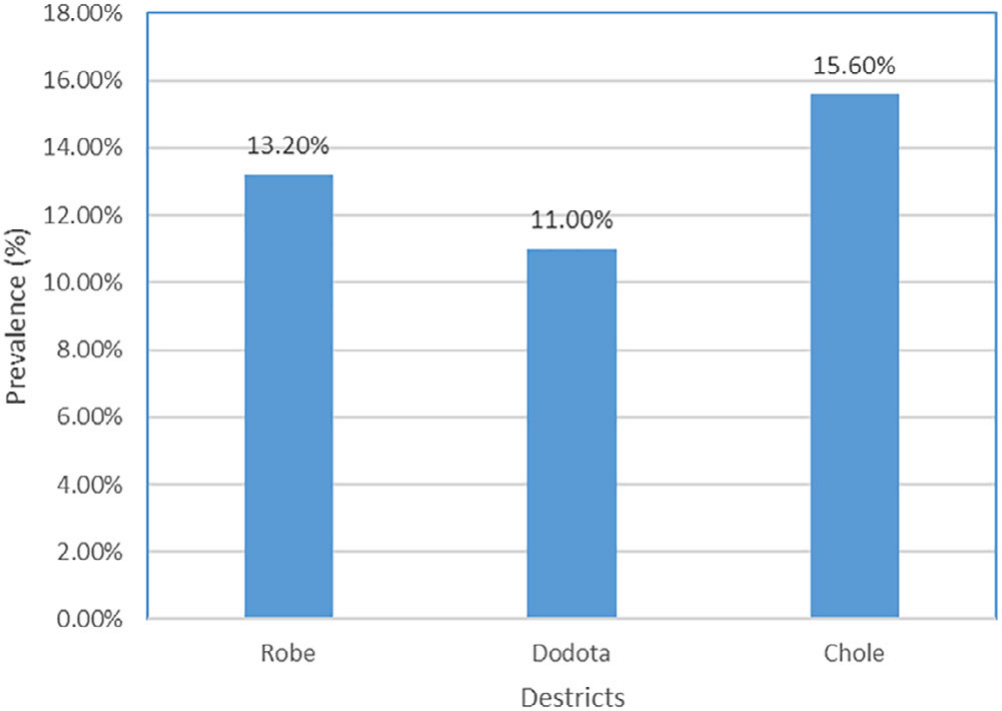
Prevalence of bovine cysticercosis by district (*n* = 510).

**TABLE 1:** Univariable and multivariable binary logistic regression analyses of bovine cysticercosis (*n* = 510) in Dodota, Robe and Chole municipal abattoirs.

Factors	Categories	No examined	No positive (%)	95% CI	cOR (95% CI)	*p* value	aOR (95% CI)	*p* value
District	Robe	250	33 (13.2)	9.56–17.96	Ref			
	Dodota	100	11 (11.0)	6.25–18.63	0.81 (0.39–1.68)	0.575		
	Chole	160	25 (15.6)	10.81–22.05	1.22 (0.69–2.14)	0.492		
Breed	Local	457	62 (13.6)	10.73–17.01				
	Cross	53	7 (13.2)	6.55–24.84	0.96 (0.42–2.24)	0.942		
Sex	Male	396	54 (13.6)	10.60–17.37	Ref			
	Female	114	15 (13.2)	8.14–20.58	0.96 (0.52–1.77)	0.895		
Age group	Young	114	9 (7.9)	4.21–14.33	Ref		Ref	
	Adult	364	42 (11.5)	8.65–15.23	1.52 (0.72–3.23)	0.274	1.57 (0.73–3.34)	0.252
	Old	32	18 (56.3)	39.33–71.83	15.0 (5.66–39.78)	<0.001	16.13 (5.88–44.21)	<0.001
Body condition	Poor	49	18 (36.7)	24.67–50.73	Ref		Ref	
	Medium	156	20 (12.8)	8.45–18.97	0.25 (0.12–0.53)	<0.001	0.25 (0.11–0.56)	<0.001
	Good	305	31 (10.2)	7.25–14.07	0.19 (0.10–0.39)	<0.001	0.18 (0.09–0.38)	<0.001
Overall prevalence		510	69 (13.5)	10.83–16.77				

*Note*: The overall fitness of the final model (Hosmer‒Lemeshow test), *χ* ^2^ = 0.07, DF = 4, *p *= 0.999. Variables with *p *< 0.25 in the univariable analysis were entered into the multivariable model; blank cells indicate variables not included in the final model.

Abbreviations: aOR, adjusted odds ratio; CI, confidence interval; cOR, crude odds ratio; DF, degree of freedom for the chi‐square test; Ref, reference group; *χ* ^2^, chi‐square test.

#### 3.1.1 Risk Factors for the Prevalence of Bovine Cysticercosis

On the basis of the univariable analysis, two factors, body condition and age group, met the screening criteria (*p* < 0.25) and were included in the multivariable logistic regression analysis. Body condition and age were statistically associated with the prevalence of bovine cysticercosis (*p* < 0.05). Thus, old cattle had approximately 16 times the odds of cysticercosis (aOR = 16.13, 95% CI: 5.88–44.21) compared with young cattle. Additionally, cattle with a medium body condition score had a 75% lower odds of developing the disease compared to poor body condition (aOR = 0.25, 95% CI: 0.11–0.56). Furthermore, cattle with good body condition scores had a 82% lower odds of developing bovine cysticercosis relative to those with poor body conditions (aOR = 0.18, 95% CI: 0.09–0.38) (Table 1).

#### 3.1.2 The Anatomical Distribution and Viability of *C. bovis*

During the detailed post‐mortem inspection at the abattoirs, a total of 142 cysts were recovered in 69 C. bovis–positive cattle. Among these 142 cysts, 40 (28.2%) were viable (live), and 102 (71.8%) were nonviable (dead). With respect to the anatomical distribution of C. bovis, the highest number of infections was found in the tongue with 37 (26.1%) cysts, followed by the heart, 26 (18.3%), and the masseter muscle, 24 (16.9%) (Table 2).

**TABLE 2:** Predilection site and viability of *Cysticercus bovis* detected in Dodota, Robe and Chole abattoirs.

Inspected organs	Total number of cysts (%)	Number of viable cysts (%)	Number of dead cysts (%)
Tongue	37 (26.1)	11 (27.5)	26 (25.5)
Masseter	24 (16.9)	6 (15.0)	18 (17.7)
Diaphragm	18 (12.7)	5 (12.5)	13 (12.8)
Shoulder	8 (5.6)	4 (10.0)	4 (3.9)
Triceps	10 (7.1)	4 (10.0)	6 (5.9)
Liver	17 (11.9)	3 (7.5)	14 (13.7)
Heart	26 (18.3)	6 (15.0)	20 (19.6)
Kidney	2 (1.4)	1 (2.5)	1 (1.0)
Total	142 (100.0)	40 (28.2)	102 (71.8)

### 3.2 The Questionnaire Survey

#### 3.2.1 Respondents’ Socio‐Demographic Characteristics

A total of 422 individuals were interviewed to assess community practices and risk perceptions regarding taeniasis. Approximately one‐third of the respondents (33.64%) were residents of the Robe district. The majority of participants (58.06%) were male, and 70.38% were married. Less than half of the respondents (45.74%) came from rural areas. Nearly half (48.10%) of the participants were between the ages of 21 and 45, and 23.00% completed a certificate programme or higher (Table 5).

#### 3.2.2 Respondents Practice Towards Taeniasis

The majority of study participants (97.39%) had access to a toilet, and most respondents (68.01%) reported using it for defecation consistently. However, only 8.29% of respondents used PPE when handling sick animals. Additionally, less than one‐third of the respondents (31.28%) deworm their animals regularly. Notably, around three‐fourths (75.59%) of the respondents consumed raw or undercooked meat, such as *kurt* and *kitfo* (Table 3).

**TABLE 3:** Respondents responses for practice‐related questions in Dodota, Robe and Chole towns.

S. no.	Questions related to community practice on taeniasis (*n* = 422)	Yes, *N* (%)
Q_1_	Do you have access to a toilet or latrine at home?	411 (97.39)
Q_2_	Do you always use the toilet for defecation (not open fields)?	287 (68.01)
Q_3_	Do you always wash hands with soap after using the toilet?	179 (42.42)
Q_4_	Do you always wash hands with soap before and after food?	294 (69.67)
Q_5_	Do you use PPE (gloves) when handling sick animals?	35 (8.29)
Q_6_	Do you deworm livestock regularly (every 3–6 months)?	132 (31.28)
Q_7_	Have you received training on taeniasis or foodborne zoonoses?	29 (6.87)
Q_8_	Do you wash meat properly before cooking?	117 (27.73)
Q_9_	Do you maintain hygiene during food preparation (clean surfaces)?	302 (71.56)
Q_10_	Do you treat drinking water (boil or filter) before consumption?	92 (21.80)
Q_11_	Do you always cook or boil meat thoroughly before eating?	74 (17.54)
Q_12_	Do you refrigerate meat promptly after purchase (slaughter)?	181 (42.89)
Q_13_	Do you prefer home (backyard) slaughter over abattoir?	341 (80.81)
Q_14_	Do you participate in group meat sharing (Kircha)?	401 (95.02)
Q_15_	Do you consume raw or undercooked meat (kitfo or kurt)?	319 (75.59)
Q_16_	Have you sold meat from sick animals to others?	30 (7.10)
Q_17_	Do you slaughter and consume meat from diseased animals?	25 (5.92)
Q_18_	Do you take preventive (prophylactic) drugs for taeniasis?	63 (14.93)
Q_19_	Do you seek medical care when infected with taeniasis?	97 (22.99)
Q_20_	Do you advise family or friends against eating raw meat?	108 (25.59)

#### 3.2.3 Study Participants’ Risk Perception Towards Taeniasis

Among 422 respondents, 49.05% perceived taeniasis as a serious public health threat in Ethiopia, whereas 36.02% believed that cysticercosis poses a significant risk to animal health. More than half of the respondents (57.11%) recognized that consuming raw or undercooked meat can lead to taeniasis. Additionally, 44.08% of respondents felt that the life cycle of *C. bovis* is sustained by open defecation. Furthermore, the majority of respondents (81.99%) did not believe that slaughtering animals at an abattoir helps reduce the risk of taeniasis (Table 4).

**TABLE 4:** Respondents risk perception towards taeniasis in Dodota, Robe and Chole towns.

S. no.	Questions related to respondents’ risk perception of taeniasis (*n* = 422)	Yes, *N* (%)
Q_1_	Taeniasis is a serious public health threat in Ethiopia	207 (49.05)
Q_2_	Cysticercosis severely threatens animal health	152 (36.02)
Q_3_	Cysticercosis severely threatens meat quality	187 (44.31)
Q_4_	Farmers, butchers and you face higher risk of taeniasis infection	190 (45.02)
Q_5_	Untreated sick animals may pose public health risks	233 (55.21)
Q_6_	Eating raw or undercooked beef may cause taeniasis	241 (57.11)
Q_7_	Open defecation maintains the *Cysticercus bovis* (taeniasis) life cycle	186 (44.08)
Q_8_	Cooking or boiling meat thoroughly reduces taeniasis risk	203 (48.10)
Q_9_	Chilling or refrigerating meat reduces taeniasis risk	117 (27.73)
Q_10_	Slaughtering animals at abattoir lowers taeniasis (cysticercosis) risk	76 (18.01)

#### 3.2.4 Overall Practice and Risk Perception Towards Bovine Cysticercosis and Taeniasis

From the total respondents, around one‐fourth (23.93%) had appropriate practices, whereas around one‐third (34.83%) had desirable risk perception towards bovine cysticercosis and taeniasis (Figure 3).

**FIGURE 3:**
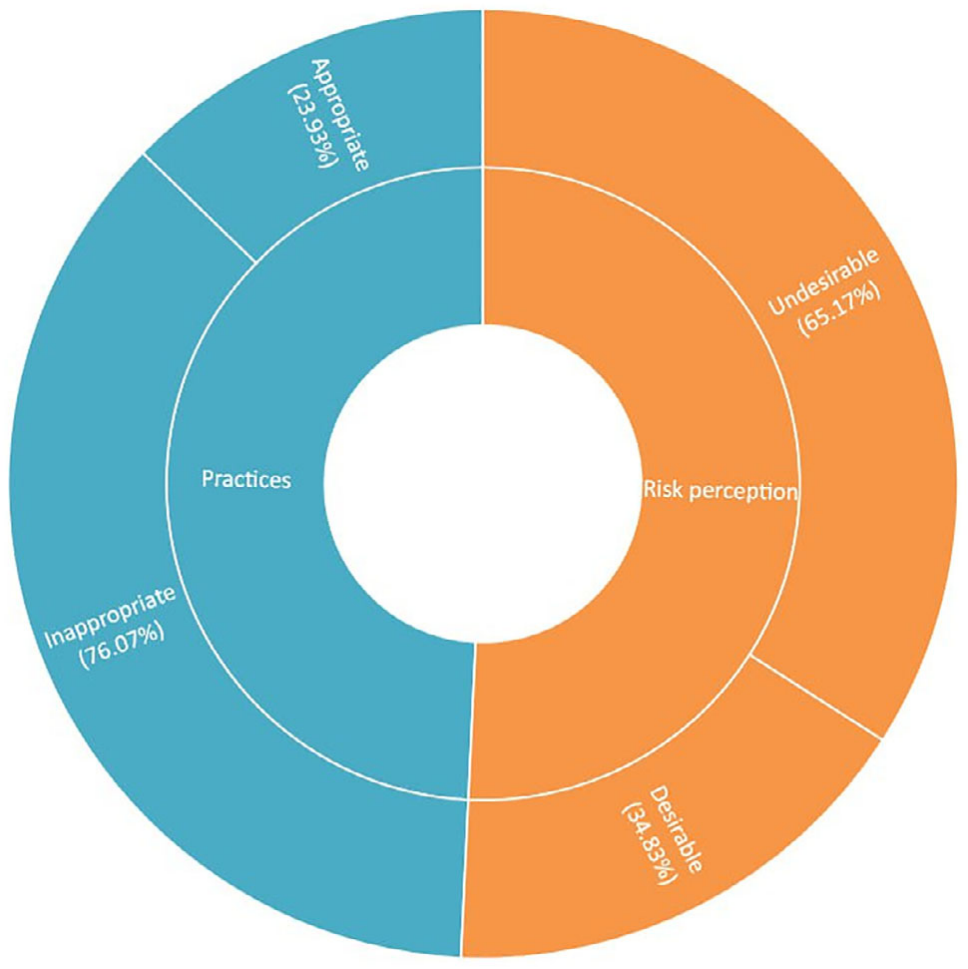
Respondents’ level of total practice and risk perception towards bovine cysticercosis and *Taenia saginata* taeniasis in Dodota, Robe and Chole towns.

#### 3.2.5 Association of Demographic Factors With Respondents’ Practice

Among the socio‐demographic variables considered, sex, religion, age group, origin and educational status were significantly associated with appropriate practices (*p* < 0.05), whereas district or town did not show such associations (Table 5). Females were 53% less likely to engage in appropriate practices compared to males (OR = 0.47, 95% CI: 0.29, 0.76). Christians had 76% higher odds of appropriate practices compared to Muslims (OR = 1.76, 95% CI: 1.12, 2.77). Those from rural origins were found to have a 59% less likelihood of appropriate practices compared to their urban counterparts (OR = 0.41, 95% CI: 0.25, 0.66). Additionally, respondents with an education level of elementary school or less were 84% less likely to practice appropriately (OR = 0.16, 95% CI: 0.08, 0.29), whereas those who completed high school were 57% less likely (OR = 0.43, 95% CI: 0.25, 0.75), both compared to individuals who achieved a diploma or higher. Furthermore, respondents aged 15–20 and those over 45 years old were found 57% (OR = 0.43, 95% CI: 0.21, 0.87) and 70% (OR = 0.30, 95% CI: 0.17, 0.51) less likely, respectively, to engage in appropriate practices related to cysticercosis and taeniasis compared to those aged 21–45 (Table 5).

**TABLE 5:** The association between communities’ socio‐demographic characteristics with their practice and perception towards *Cysticercus bovis* (taeniasis) in Dodota, Robe and Chole towns (*n* = 422).

Variables	Categories	*N* (%)	*N* positive practice (%)	aOR (95% CI)	*p* value	*N* desirable perception (%)	aOR (95% CI)	*p* value
Town or District	Robe	142 (33.64)	40 (28.17)	ref		63 (44.37)	Ref	
	Dodota	140 (33.18)	33 (23.57)	0.79 (0.46, 1.34)	0.1893	48 (34.29)	0.65 (0.40, 1.06)	0.0519
	Chole	140 (33.18)	28 (20.00)	0.64 (0.37, 1.11)	0.0551	36 (25.71)	0.43 (0.26, 0.72)	0.0006
Sex	Male	245 (58.06)	72 (29.39)	ref		96 (39.18)	Ref	
	Female	177 (41.94)	29 (16.38)	0.47 (0.29, 0.76)	0.0011	49 (27.68)	0.59 (0.39, 0.90)	0.0073
Religion	Christian	193 (45.73)	57 (29.53)	1.76 (1.12, 2.77)	0.0139	76 (39.38)	1.45 (0.97, 2.16)	0.0726
	Muslim	229 (54.27)	44 (19.21)	Ref		71 (31.00)	Ref	
Age group in years	15–20	61 (14.46)	11 (18.03)	0.43 (0.21, 0.87)	0.0098	17 (27.87)	0.67 (0.36, 1.26)	0.1089
	21–45	203 (48.10)	69 (33.99)	Ref		74 (36.45)	Ref	
	>45	158 (37.44)	21 (13.29)	0.30 (0.17, 0.51)	<0.0001	56 (35.44)	0.96 (0.62, 1.48)	0.4214
Origin	Rural	193 (45.74)	30 (15.54)	0.41 (0.25, 0.66)	0.0001	52 (26.94)	0.52 (0.34, 0.79)	0.0009
	Urban	229 (54.26)	71 (31.00)	Ref		95 (41.48)	Ref	
Education level	Elementary and less	173 (41.00)	19 (10.98)	0.16 (0.08, 0.29)	<0.0001	46 (26.59)	0.42 (0.25, 0.71)	0.0005
	High school	152 (36.00)	39 (25.66)	0.43 (0.25, 0.75)	0.0012	56 (36.84)	0.67 (0.40, 1.13)	0.0676
	Diploma and above	97 (23.00)	43 (44.33)	Ref		45 (46.39)	Ref	

Abbreviations: aOR, adjusted odds ratio; CI, confidence interval; *N*, number; Ref, reference.

#### 3.2.6 Association of Demographic Factors With Respondents’ Risk Perception

Regarding the respondents' risk perception, sex, educational status, origin and district had significant associations with desired risk perception towards cysticercosis and taeniasis (*p* < 0.05) (Table 5). Female respondents had 41% (OR = 0.59, 95% CI: 0.39, 0.90) lower odds of desirable risk perception than males. Respondents who had completed elementary school or less were 58% less likely to exhibit desired risk perception towards cysticercosis and taeniasis (OR = 0.42, 95% CI: 0.25, 0.71). Rural residents may have a lower odds of desired risk perception than urban residents (OR = 0.52, 95% CI: 0.34, 0.79). Furthermore, Christian respondents revealed a 45% more likely to have a desired risk perception towards cysticercosis and taeniasis compared to Muslims (OR = 1.45, 95% CI: 0.97, 2.16). Respondents found in Chole district have also less odds of desirable risk perception by 57% (OR = 0.43, 95% CI: 0.26, 0.72) compared to residents in Robe (Table 5).

## 4 Discussion

The current study revealed a moderately high prevalence of bovine cysticercosis, coupled with significantly low community preventive practices and poor risk perception towards bovine cysticercosis and human taeniasis. The total prevalence of bovine cysticercosis was 13.5% (95% CI: 10.8–16.8). This finding aligns closely with previous epidemiological reports from various regions in Ethiopia, including Wolaita Soddo (11.3%; Alemayehu et al. 2009), Yirgalem (12.0%; Abunna 2013) and Haramaya (14.4%; Abdurehman et al. 2020).

However, the prevalence observed here is higher than those reported in other Ethiopian localities, such as Addis Ababa (2.8%; Mohamed et al. 2021 and 3.6%; Ibrahim and Zerihun 2012), Nekemte (2.9%; Shuramo et al. 2022), Jimma (4.4%; Megersa et al. 2010), Bishoftu (4.2%; Fesseha and Asefa 2023 and 7.8%; Tariku and Beredo 2019), Bahir Dar (4.2%; Birhanu et al. 2018), Debre Berhan (4.6%; Kifle and Belete 2015), Kombolcha (6.4%; Engdaw et al. 2015) and Dessie (9.6%; Yigizaw et al. 2017). Internationally, our observed rates exceed estimates reported in Egypt (0.34%–5.70%; El‐Dakhly et al. 2023), Botswana (6.2%; Mazhani et al. 2022), Europe (0.41%–14.0%; Laranjo‐González et al. 2016), Iran (0.25%; Khaniki et al. 2009) and Brazil (0.01%–18.75%; Rossi et al. 2020). Conversely, higher infection rates have been documented elsewhere in Ethiopia, including Nekemte (21.6%; Tsegaye et al. 2026), Adama (19.7%; Birhanu and Abda 2014), Hawassa (22.9%; Belachew and Ibrahim 2012), Meki (30.7%; Edao and Korso 2019) and Silte Zone (36.0%; Musema 2023).

These spatial discrepancies in prevalence likely stem from a complex interplay of host, environmental and methodological factors (Tsegaye et al. 2026). Variations in agro‐ecological conditions, local human behavioural patterns regarding raw meat consumption, sample size choices, animal management systems and cattle origins for slaughter all contribute to observed heterogeneity. Furthermore, variation in routine post‐mortem examination techniques or diagnostic sensitivity, sampling design, slaughter populations, study periods and case definitions of cysticercosis inherently underestimate true prevalence. Meat inspection protocols enforce legal limits on the depth and number of diagnostic incisions permitted, as excessive mutilation impairs carcass marketability (Birhanu and Abda 2014; Abdurehman et al. 2020). Consequently, light‐to‐moderate cyst workloads frequently evade standard detection.

In this study, cattle with poor body condition exhibited significantly higher odds of harbouring *C. bovis* larvae, consistent with earlier investigations (Yigizaw et al. 2017; Netsanet et al. 2020; Musema 2023). Poor body condition may reflect chronic parasitemia, poor feed conversion efficiency or underlying nutritional stress that dampens host immunity. In contrast, Birhanu and Abda (2014) documented higher prevalence among well‐conditioned animals. This variation is primarily attributable to husbandry regimes: Cattle in intensive fattening feedlots are typically managed under semi‐zero or zero‐grazing conditions, tethered in controlled environments, provided non‐contaminated feed and water and routinely dewormed (Netsanet et al. 2020), thereby reducing their exposure to pasturelands contaminated with *T. saginata* oncospheres.

Age was similarly identified as an important epidemiological variable influencing infection status, in agreement with existing literature (Engdaw et al. 2015; Kifle and Belete 2015; Edao and Korso 2019; Abdurehman et al. 2020). Older animals demonstrated a significantly higher susceptibility to cysticercosis than younger cattle, corroborating findings by Birhanu and Abda (2014) and Edao and Korso (2019). This positive correlation between age and prevalence is likely a function of cumulative lifetime exposure to contaminated pastures and water sources (Tsegaye et al. 2026). Conversely, some studies have noted higher infection rates in younger stock (Abdurehman et al. 2020), whereas several others identified no statistically significant association between age and infection (Abunna 2013; Yigizaw et al. 2017; Birhanu et al. 2018; Netsanet et al. 2020; Mohamed et al. 2021; Musema 2023). Such conflicts underscore regional variations in animal acquisition, age‐dependent immune responses and local slaughterhouse demographics (Abdurehman et al. 2020).

Although *C. bovis* lacks strict tissue tropism, the anatomical distribution of metacestodes varies markedly between organs (Scandrett et al. 2009). The tongue and heart were the primary and secondary sites of preference in this investigation, followed by the masseter muscles. This anatomical distribution aligns with documented infestation patterns in Ethiopia (Kifle and Belete 2015; Birhanu et al. 2018; Edao and Korso 2019; Geinoro and Bedore 2019; Abdurehman et al. 2020; Netsanet et al. 2020), although some reports highlight the heart as the primary site (Regassa et al. 2009; Birhanu et al. 2018). The predilection for the masticatory apparatus (tongue and masseters) is physiological: These highly active muscles receive robust arterial perfusion during grazing and rumination, facilitating oncosphere lodging (Scandrett et al. 2009). Moreover, routine regulatory inspection permits deep, multiple incisions into the masseters and tongue without substantially degrading carcass retail value; diagnostic sensitivity is naturally higher for these tissues relative to primary prime‐cut skeletal muscles (Scandrett et al. 2009; Shuramo et al. 2022).

Regarding cyst degeneration, a higher proportion of calcified or degenerative (dead) cysts was recovered relative to viable (fertile) metacestodes. This finding is consistent with nationwide trends (Regassa et al. 2009; Birhanu and Abda 2014; Geinoro and Bedore 2019; Abdurehman et al. 2020; Netsanet et al. 2020; Mohamed et al. 2021), though Birhanu et al. (2018) reported a higher frequency of viable cysts. Divergences in viable‐to‐calcified cyst ratios across studies reflect differences in host immune‐mediated cyst degradation, inspector experience, diagnostic slicing density and the latency period between initial infection and slaughter (Regassa et al. 2009).

Epidemiological maintenance of the *T. saginata* life cycle relies directly on human behaviours that sustain environmental contamination and facilitate parasite ingestion. A critical finding of this study is the disconnection between perceived health risk and poor personal sanitation and dietary practices. Approximately 75% of surveyed participants reported consuming raw or undercooked beef (*kitfo* and *kurt*), and a clear majority favoured uninspected backyard slaughter over municipal abattoirs. These figures mirror national behavioural data indicating high raw meat consumption (78.3%–85.3%) and widespread backyard slaughter (55.8%–89.5%) in Ethiopia (Jorga et al. 2022; Tsegaye et al. 2026).

Compounding this risk, roughly one‐third of respondents reported practicing open defecation near pastures, roadsides and water bodies when away from home, despite possessing latrine infrastructure at home. Furthermore, over one‐third neglected basic hand hygiene with soap post‐defecation. Similar behavioural paradoxes have been recorded elsewhere in Ethiopia; for instance, Ambaw (2025) observed that despite 100% household latrine coverage in Maya City, 64.2% of residents continued open defecation in outdoor spaces. These open defecation practices contaminate environmental water bodies and communal pastures with infectious *T. saginata* eggs, particularly during seasonal flooding events (Rossi et al. 2020; Fenta et al. 2025).

Socio‐demographic stratification yielded distinct behavioural trends across the population. Over 75% of individuals experiencing tapeworm infection did not seek formal medical care, preferring self‐medication or non‐intervention, a pattern that mirrors the 84.2% non‐treatment‐seeking rate documented by Tsegaye et al. (2026). Religious and cultural traditions also structured these exposure pathways: Christian respondents displayed higher rates of preventative practices than Muslim respondents, as culturally embedded traditions, including raw beef consumption during major religious festivals (e.g., Ethiopian New Year) and informal meat‐sharing schemes (*kircha*), serve as key operational drivers of transmission (Seleshe et al. 2014; Jorga et al. 2022; Tsegaye et al. 2026).

Along the urban–rural axis, rural residency was significantly associated with suboptimal preventative practices compared to urban residency (Tsegaye et al. 2026). However, higher urban residency and household income increase purchasing power for raw beef, paradoxically expanding exposure pathways within city centres (Betru and Kawashima 2009; Jorga et al. 2022). Regarding gender, female respondents demonstrated lower disease awareness and practice scores than male respondents (Bibi et al. 2023), contrasting with studies reporting equal practice levels across sexes (Jorga et al. 2022; Tsegaye et al. 2026). In Ethiopian society, men disproportionately engage in livestock slaughter, butcher transactions and social raw meat consumption, leading to higher experiential awareness of infection risks (Adem and Mohammed 2019; Jorga et al. 2022). Higher educational status (diploma or above) was strongly correlated with safe hygienic behaviours and a preference for thoroughly cooked meat (Regassa et al. 2009; Jorga et al. 2022; Bibi et al. 2023), underscoring education as an effective structural intervention. Age‐wise, risky dietary behaviours peaked among young adults (21–45 years), likely reflecting economic independence and higher frequency of dining outside the home (Tsegaye et al. 2026; Jorga et al. 2022).

Addressing the endemic burden of bovine cysticercosis and human taeniasis requires an integrated One Health framework uniting veterinary health, environmental management and public health sectors. First, environmental contamination must be minimized by eliminating open defecation pathways, expanding rural sanitation access and enforcing agricultural containment measures that prevent livestock grazing on contaminated floodplains and pasturelands (Rossi et al. 2020; Fenta et al. 2025). Second, regulatory authorities must enforce strict prohibitions against illicit backyard slaughter while standardizing diagnostic protocols in official abattoirs (Phumrattanaprapin et al. 2024; Tsegaye et al. 2026). Meat processing facilities should adopt Hazard Analysis and Critical Control Point (HACCP) principles to systematically identify and isolate infected carcasses prior to commercial distribution (Fenta et al. 2025). Third, health systems should ensure access to effective anthelmintics (e.g., praziquantel and niclosamide) for human taeniasis cases (Lateef et al. 2008). Given emerging reports of treatment failures and anthelmintic resistance in cestodes, active pharmacovigilance and research into alternative therapeutic candidates remain essential (Lateef et al. 2008; Márquez‐Navarro et al. 2012; Mohanty et al. 2017). Finally, culturally tailored health education interventions must target high‐risk demographics, school‐aged children, farmers, meat handlers and food vendors, emphasizing parasite life‐cycle dynamics, safe slaughter practices, proper handwashing and the health risks associated with consuming raw or undercooked beef (Jorga et al. 2022; Tsegaye et al. 2026).

## 5 Limitations of the Study

The first limitation was that parasitological identification relied on routine post‐mortem meat inspection, morphological examination and scolex evagination tests without molecular (PCR) or serological (ELISA) verification. Because routine inspection omits unexamined tissues and exhibits low diagnostic sensitivity, the true prevalence of *C. bovis* is likely underestimated. Second, the cross‐sectional study design precludes establishing causal relationships between the identified risk factors and *C. bovis* infection. Third, potential sampling bias cannot be completely excluded due to the absence of a comprehensive sampling frame at the abattoir level. Fourth, the community questionnaire sample size was calculated without applying a design effect multiplier for the multistage cluster sampling design; unaccounted intra‐cluster correlation across kebeles and villages may have artificially narrowed CIs and inflated statistical precision. Finally, survey responses regarding risk perceptions and meat consumption behaviours may be subject to social desirability or recall bias.

## 6 Conclusion

This study demonstrated a moderately high prevalence of bovine cysticercosis together with poor community practices and negative risk perception, which highlights the need for a resilient disease prevention and control programme in the study areas. The tongue, heart, master muscle and diaphragm were the commonly reported sites of bovine cysticercosis. Age group and body condition score were potential predictors for the prevalence of bovine cysticercosis, but sex, origin and breed were not. Respondents’ sex, origin and educational status were also significant factors for better practice and risk perception. Therefore, special attention is encouraged during meat inspection of poorly body‐conditioned and aged animals in abattoirs. Furthermore, it is essential to enhance public education efforts to discourage the consumption of raw meat, promote proper sanitation practices and underscore the necessity for collaboration among diverse stakeholders within a One Health framework.

## Author Contributions

**Yihenew Getahun Ambaw**: conceptualization, methodology, data curation, formal analysis, writing – original draft. **Ahmed Jemalo Jarso**: conceptualization, methodology, data collection, writing – original draft. **Ambachew Motbaynor Wubaye**: methodology, formal analysis, visualization, writing – review and editing. **Adem Hiko**: writing – original draft, writing – review and editing, supervision. **Animaw Andargie Worku**: data collection, writing – review and editing. **Assaye Wollelie Fentie** : data collection, writing – review and editing. **Simegnew Adugna Kallu**: methodology, formal analysis, writing – review and editing.

## Funding

The authors have nothing to report.

## Ethics Statement

For this investigation, ethical approval was obtained from Haramaya University Research and Ethical Review Committee (Ref. No: HU/548/2022). Informed consent was obtained from all human study participants, who demonstrated their agreement to participate by signing consent forms; participation was entirely voluntary. All animal handling and management were conducted by veterinarians following international ethical standards for the humane care of animals.

## Consent

Written informed consent for publication of their survey details or results was obtained from the respondents.

## Conflicts of Interest

The authors declare no conflicts of interest.

## Supporting information




**Supporting File 1**: vms371317‐sup‐0001‐S1 file.docx

## Data Availability

All the data used for analysis in this study are available from the corresponding author upon reasonable request.

## References

[vms371317-bib-0001] Abdurehman, A. , S. Hussen , M. Jafer , F. Aliye , A. Jemal , and S. Abdusemed . 2020. “ *Cysticercus bovis*: Prevalence, Associated Risk Factorsand Cyst Character per Organ Load in Haramaya Municipalabattoireast Hararghe Zone of Oromia Regional State, Ethiopia.” Journal of Microbial and Biochemical Technology 12, no. 5: 438.

[vms371317-bib-0002] Abunna, F. 2013. “Prevalence, Organ Distribution, Viability and Socioeconomic Implication of Bovine Cysticercosis/Teniasis, Ethiopia.” Revue D Elevage Et De Medecine Veterinaire Des Pays Tropicaux 66, no. 1: 25–30. file:///C:/Users/melke/Downloads/dbastia,+REMVT13_025_030%20(1).pdf.

[vms371317-bib-0003] Abunna, F. , G. Tilahun , B. Megersa , A. Regassa , and B. Kumsa . 2018. “Bovine Cysticercosis in Cattle Slaughtered at Awassa Municipal Abattoir, Ethiopia: Prevalence, Cyst Viability, Distribution and Its Public Health Implication.” Zoonoses Public Health 55, no. 2: 82–88. 10.1111/j.1863-2378.2007.01091.x. 18234026

[vms371317-bib-0004] Adem, G. , and S. Mohammed . 2019. “Causes of Gender Division of Labour Among Husbands and Wives in Bale Agro‐Pastoralist Woredas.” LJRHSS 19: 216–224. https://journalspress.com/causes‐of‐gender‐division‐of‐labour‐among‐husbands‐and‐wives‐in‐bale‐agro‐pastoralist‐woredas/.

[vms371317-bib-0005] Alemayehu, R. , A. Hailu , F. Gedissa , and M. Biniyam . 2009. “Cysticercosis of Slaughtered Cattle in Wolaita Soddo, Ethiopia.” Research in Veterinary Science 47: 322–346.

[vms371317-bib-0006] Ambaw, Y. G. 2025. “Cattle Schistosomiasis With Farmers′ Practice Regarding the Prevention and Control of Zoonotic Trematods in Maya City, Eastern Ethiopia.” Journal of Parasitology Research 2025: 1–11. 10.1155/japr/7602193. PMC12767077 41497324

[vms371317-bib-0007] Arsi Zone Agriculture Office (AZAO) . 2023. Livestock and Fishery Sector.

[vms371317-bib-0008] Belachew, M. , and N. Ibrahim . 2012. “Prevalence of *Cysticercus bovis* in Hawassa Municipal Abattoir and Its Public Health Implication.” AEJSR 7, no. 6: 238–245. 10.5829/idosi.aejsr.2012.7.6.6565.

[vms371317-bib-0009] Betru, S. , and H. Kawashima . 2009. “Pattern and Determinants of Meat Consumption in Urban and Rural Ethiopia.” LRRD 21, no. 9. https://www.lrrd.cipav.org.co/lrrd21/9/betr21143.htm.

[vms371317-bib-0010] Bibi, S. , M. Kamran , H. Ahmad , et al. 2023. “Knowledge, Attitudes and Practices Regarding Taeniasis in Pakistan.” Diseases (Basel, Switzerland) 11, no. 3: 95. 10.3390/diseases11030095. PMC10366766 37489447

[vms371317-bib-0011] Birhanu, T. , and S. Abda . 2014. “Prevalence, Economic Impact and Public Perception of Hydatid Cyst and *Cysticercus bovis* on Cattle Slaughtered at Adama Municipal Abattoir, South Eastern Ethiopia.” Journal of Scientific Research 9, no. 4: 87–97.

[vms371317-bib-0012] Birhanu, T. , T. Habtamu , and G. Mu‐uz . 2018. “Prevalence, Financial Impact and Public Health Significance of *Cysticercus bovis* at Bahir Dar Municipal Abattoir, Ethiopia.” Journal of Veterinary Medicine and Animal Health 10, no. 1: 14–20. 10.5897/JVMAH2017.0650.

[vms371317-bib-0013] Edao, L. , and A. Korso . 2019. “Prevalence of *Cysticercosis bovis* in Eastern Shoa of Oromia, Ethiopia.” Journal of Biology, Agriculture and Healthcare 9, no. 7: 58.

[vms371317-bib-0014] El‐Dakhly, K. M. , S. A. Hany , W. M. Arafa , O. R. Abdel‐Fatah , N. S. Abdel‐Atty , and E. S. El‐Nahass . 2023. “The Prevalence and Molecular Detection of Bovine Cysticercosis and Its Impact on Slaughtered Cattle in Egypt.” Journal of Parasitic Diseases 47, no. 3: 527–534. 10.1007/s12639-023-01596-5. PMC10382426 37520208

[vms371317-bib-0015] Engdaw, T. A. , A. T. Alemneh , and S. T. Ambaw . 2015. “Study on the Prevalence of *Cysticercus bovis* in Kombolcha Elfora, North‐Eastern Ethiopia.” European Journal of Applied Sciences 7, no. 4: 152–157. 10.5829/idosi.ejas.2015.7.4.96109.

[vms371317-bib-0016] Fenta, M. D. , B. Eshetu , A. B. Bitew , H. Dejene , and M. Z. Kinde . 2025. “Foodborne and Neglected Parasitic Zoonoses in Ethiopian Red Meat Animals: Insights From a Systematic Review and Meta‐Analysis.” Frontiers in Veterinary Science 12: 1501940. 10.3389/fvets.2025.1501940. PMC12173893 40534785

[vms371317-bib-0017] Fesseha, H. , and I. Asefa . 2023. “Prevalence and Associated Risk Factors of *Cysticercosis bovis* in Bishoftu Municipal Abattoir, Central Ethiopia.” Environ Health Insights 17: 11786302231164298. 10.1177/11786302231164298. PMC10052585 37007220

[vms371317-bib-0018] Geinoro, T. , and B. Bedore . 2019. “Prevalence of *Cysticercus bovis* in Cattle Slaughtered at Bishoftu Municipal Abattoir; Public Health Significance and Community Perception About Zoonotic Importance of Taeniosis in Bishoftu.” International Journal of Advanced Research in Biological Sciences 6, no. 4: 52–61. 10.22192/ijarbs.2019.06.04.008.

[vms371317-bib-0019] Hosmer, D. W. , S. Lemeshow , and R. X. Sturdivant . 2013. Applied Logistic Regression. 3rd ed. John Wiley & Sons. 10.1002/9781118548387.

[vms371317-bib-0020] Ibrahim, N. , and F. Zerihun . 2012. “Prevalence of *Tania saginata* Cysticercosis in Cattle Slaughtered in Addis Ababa Municipal Abattoir, Ethiopia.” Global Veterinaria 8, no. 5: 467–471.

[vms371317-bib-0021] James, G. , D. Witten , T. Hastie , and R. Tibshirani . 2013. An Introduction to Statistical Learning: With Applications in R. Springer. 10.1007/978-1-4614-7138-7.

[vms371317-bib-0022] Jorga, E. , I. Van Damme , B. Mideksa , and S. Gabriël . 2020. “Identification of Risk Areas and Practices for *Taenia saginata* Taeniosis/Cysticercosis in Ethiopia: A Systematic Review and Meta‐Analysis.” Parasites & Vectors 13, no. 1: 375. 10.1186/s13071-020-04222-y. PMC7391523 32727549

[vms371317-bib-0023] Jorga, E. , I. Van Damme , B. Mideksa , and S. Gabriël . 2022. “Knowledge, Attitude, and Practices of the Community and Meat Industry Workers Towards *Taenia saginata* Taeniosis and Bovine Cysticercosis in and Around Jimma and Ambo Towns of Ethiopia.” Preventive Veterinary Medicine 204: 105653. 10.1016/j.prevetmed.2022.105653. 35500336

[vms371317-bib-0024] Khaniki, G. R. , M. Raei , E. B. Kia , A. M. Haghi , and M. Selseleh . 2009. “Prevalence of Bovine Cysticercosis in Slaughtered Cattle in Iran.” Tropical Animal Health and Production 42, no. 2: 141–143. 10.1007/s11250-009-9399-3. 19568950

[vms371317-bib-0025] Kifle, W. , and S. Belete . 2015. “Prevalence and Public Health Significance of Cyst *Cercus bovis* in and Around Debre Berhan City.” European Journal of Applied Sciences 7, no. 5: 199–208. 10.5829/idosi.ejas.2015.7.5.9692.

[vms371317-bib-0026] Laranjo‐González, M. , B. Devleesschauwer , S. Gabriël , P. Dorny , and A. Allepuz . 2016. “Epidemiology, Impact and Control of Bovine Cysticercosis in Europe: A Systematic Review.” Parasites & Vectors 9: 81. 10.1186/s13071-016-1362-3. PMC4748494 26860313

[vms371317-bib-0028] Lateef, M. , S. A. Zargar , A. R. Khan , M. Nazir , and A. Shoukat . 2008. “Successful Treatment of Niclosamide‐ and Praziquantel‐Resistant Beef Tapeworm Infection With Nitazoxanide.” International Journal of Infectious Diseases: IJID: Official Publication of the International Society for Infectious Diseases 12, no. 1: 80–82. 10.1016/j.ijid.2007.04.017. 17962058

[vms371317-bib-0030] Márquez‐Navarro, A. , M. C. Cornejo‐Coria , F. Cebada‐López , R. M. Sánchez‐Manzano , D. L. Díaz‐Chiguer , and B. Nogueda‐Torres . 2012. “ *Taenia saginata*: Failure Treatment in a Child With 5‐Year Long‐Lasting Infection.” Gastroenterology Nursing: The Official Journal of the Society of Gastroenterology Nurses and Associates 35, no. 2: 125–127. 10.1097/SGA.0b013e31824eb933. 22472672

[vms371317-bib-0031] Mazhani, B. , E. Masitha , M. Ntwaetsile , K. Thutwa , and K. Sehularo . 2022. “Distribution of Bovine Cysticercosis Prevalence in the Southeastern Districts of Botswana From 2015 to 2016.” Veterinary World 15, no. 2: 368–373. 10.14202/vetworld.2022.368-373. PMC8980385 35400945

[vms371317-bib-0032] Megersa, B. , E. Tesfaye , A. Regassa , R. Abebe , and F. Abunna . 2010. “Bovine Cysticercosis in Cattle Slaughtered at Jimma Municipal Abattoir, South Western Ethiopia: Prevalence, Cyst Viability and Its Socio‐Economic Importance.” Veterinary World 3, no. 6: 257. www.veterinaryworld.org.

[vms371317-bib-0033] Ministry of Agriculture (MoA) . 2021. Meat Inspection Guidelines for Domestic Abattoirs. Ministry of Agriculture.

[vms371317-bib-0034] Mohamed, A. , M. Abebe , W. Birhanu , M. Abdirahman , and M. A. Wali . 2021. “Prevalence of *Taenia saginata* Cysticerci in Addis Ababa Abattoir Enterprise, Ethiopia.” FWP 25: e00135. 10.1016/j.fawpar.2021.e00135. PMC8521109 34703918

[vms371317-bib-0035] Mohanty, A. , T. S. Singh , T. O. Bhutia , P. Gupta , and P. Gupta . 2017. “A Case of Albendazole and Niclosamide Resistant *Taenia saginata* Infection.” International Journal of Research in Medical Sciences 5, no. 6: 2821–2823. 10.18203/2320-6012.ijrms20172497.

[vms371317-bib-0036] Musema, S. M. 2023. “Prevalence of Bovine Cysticercosis, Human Taeniasis and Asociated Risk Factors in Selected Towns of Silte Zone, Southern Ethiopia.” Applied Medical Research 10, no. 2: 1–13. 10.1101/2023.01.25.525623.

[vms371317-bib-0037] Ndlovu, T. , M. Chimonyo , A. I. Okoh , V. Muchenje , K. Dzama , and J. G. Raats . 2007. “Assessing the Nutritional Status of Beef Cattle: Current Practices and Future Prospects.” African Journal of Biotechnology 6, no. 24: 2727–2734. http://www.academicjournals.org/AJB.

[vms371317-bib-0038] Netsanet, B. G. , Y. A. Yesuf , B. H. Abrha , and B. D. Lewtnesh . 2020. “Prevalence and Public Health Importance of Bovine Cysticercosis in and Around Dessie, Amhara Region, North East Ethiopia.” NJAS 22, no. 2: 173–185. https://www.ajol.info/index.php/tjas/article/view/200528.

[vms371317-bib-0039] Nigatu, K. 2004. “Cysticercus bovis: Development and Evaluation of Serological Tests and Prevalence at Addis Ababa Abattoir.” Master of Science thesis, Addis Ababa University, Faculty of Veterinary Medicine.

[vms371317-bib-0040] Nyangi, C. , D. Stelzle , E. M. Mkupasi , et al. 2022. “Knowledge, Attitudes and Practices Related to *Taenia solium* Cysticercosis and Taeniasis in Tanzania.” BMC Infectious Diseases 22, no. 1: 534. 10.1186/s12879-022-07408-0. PMC9190087 35692033

[vms371317-bib-0041] Phumrattanaprapin, W. , N. J. Tatiya‐Apiradee , P. Antaban , and W. Mahikul . 2024. “Assessment of Knowledge and Practices Regarding Taeniasis and Cysticercosis in Pak Chong, Nakhon Ratchasima, Thailand: A Cross‐Sectional Study.” PLoS ONE 19, no. 7: e0307240. 10.1371/journal.pone.0307240. PMC11249257 39008473

[vms371317-bib-0042] Regassa, A. , F. Abunna , A. Mulugeta , and B. Megersa . 2009. “Major Metacestodes in Cattle Slaughtered at Wolaita Soddo Municipal Abattoir, Southern Ethiopia: Prevalence, Cyst Viability, Organ Distribution and Socioeconomic Implications.” Tropical Animal Health and Production 41, no. 7: 1495–1502. 10.1007/s11250-009-9338-3. 19353302

[vms371317-bib-0043] Rossi, G. A. M. , I. Van Damme , and S. Gabriël . 2020. “Systematic Review and Meta‐Analysis of Bovine Cysticercosis in Brazil: Current Knowledge and Way Forward.” Parasites & Vectors 13, no. 1: 92. 10.1186/s13071-020-3971-0. PMC7035740 32085721

[vms371317-bib-0044] Scandrett, B. , S. Parker , L. Forbes , et al. 2009. “Distribution of *Taenia saginata* Cysticerci in Tissues of Experimentally Infected Cattle.” Veterinary Parasitology 164, no. 2‐4: 223–231. 10.1016/j.vetpar.2009.05.015. 19524368

[vms371317-bib-0045] Seleshe, S. , C. Jo , and M. Lee . 2014. “Meat Consumption Culture in Ethiopia.” Food Science of Animal Resources 34, no. 1: 7–13. 10.5851/kosfa.2014.34.1.7. PMC4597829 26760739

[vms371317-bib-0046] Shuramo, M. Y. , K. P. Gutama , M. R. Bulcha , and M. Pal . 2022. “Prevalence, Related Risk Factors and Distribution of Cyst of *Cysticercus bovis* in Cattle Subjected to Slaughter at Nekemte Municipal Abattoir, Western Ethiopia.” International Journal of Livestock Research 12, no. 1: 27–32. 10.5455/ijlr.20220130050629.

[vms371317-bib-0047] Sithole, M. I. , J. L. Bekker , A. M. Tsotetsi‐Khambule , and S. Mukaratirwa . 2019. “Ineffectiveness of Meat Inspection in the Detection of *Taenia solium* Cysticerci in Pigs Slaughtered at Two Abattoirs in the Eastern Cape Province of South Africa.” Veterinary Parasitology, Regional Studies and Reports 17: 100299. 10.1016/j.vprsr.2019.100299. 31303223

[vms371317-bib-0048] Subedi, D. , A. Dhakal , S. Jyoti , et al. 2025. “Zoonotic Diseases Awareness and Food Safety Practices Among Livestock Farmers in Nepal.” Frontiers in Veterinary Science 11: 1514953. 10.3389/fvets.2024.1514953. PMC11770021 39872611

[vms371317-bib-0049] Subedi, D. , S. Jyoti , B. Thapa , et al. 2023. “Knowledge, Attitude, and Practice of Antibiotic Use and Resistance Among Poultry Farmers in Nepal.” Antibiotics (Basel, Switzerland) 12, no. 9: 1369. 10.3390/antibiotics12091369. PMC10525118 37760666

[vms371317-bib-0050] Tariku, G. , and B. Beredo . 2019. “Prevalence of *Cysticercus bovis* in Cattle Slaughtered at Bishoftu Municipal Abattoir; Public Health Significance and Community Perception About Zoonotic Importance of Taeniosis in Bishoftu.” International Journal of Advanced Research in Biological 6, no. 4: 52–61. 10.22192/ijarbs.2019.06.04.008.

[vms371317-bib-0052] Thrusfield, M. 2018. Veterinary Epidemiology. 4th ed. John Wiley & Sons. www.wiley.com/go/veterinariyepidemiology.

[vms371317-bib-0053] Torell, R. , B. Bruce , B. Kvasnicka , and K. Conley . 2003. Methods of Determining Age of Cattle. Cattle Producer's Library: CL712. University of Nevada. Accessed on 4 July, 2013. http://www.unce.unr.edu/publications/files/ag/other/cl712.pdf.

[vms371317-bib-0054] Tsegaye, D. , L. Olani , D. Shiferaw , et al. 2026. “Bovine Cysticercosis at a Municipal Abattoir: Occurrence, Risk Factors, Community Practices and Perceptions in Nakemte, Western Ethiopia.” Preventive Veterinary Medicine 254: 106942. 10.1016/j.prevetmed.2026.106942. 42330777

[vms371317-bib-0055] WHO & FAO . 2014. Multicriteria‐Based Ranking for Risk Management of Food‐Borne Parasites: report of a Joint FAO/WHO expert meeting, 3‐7 September 2012, FAO Headquarters, Rome, Italy. FAO, World Health Organization. https://iris.who.int/handle/10665/112672.

[vms371317-bib-0056] Yigizaw, G. , Y. Tefera , and T. Tintagu . 2017. “Prevalence of *Cysticercus bovis* at Dessie Municipal Abattoir, North East Ethiopia.” Abyssinia Journal of Science & Technology 2, no. 1: 25–29. https://www.ajol.info/index.php/abjst/article/view/281014.

[vms371317-bib-0057] Zulu, G. , K. E. Mwape , T. M. Welte , et al. 2023. “Community Knowledge, Attitudes and Practices Related to *Taenia solium* Taeniosis and Cysticercosis in Zambia.” PLOS Neglected Tropical Diseases 17, no. 8: e0011375. 10.1371/journal.pntd.0011375. PMC10443877 37561784

