# Supplementary material for: Bovine Cysticercosis in Dodota, Robe and Chole Municipal Abattoirs and Community Practices and Risk Perceptions Regarding Taeniasis in Ethiopia
Source: Vet Med Sci. 2026 Nov 6;12(6):e71317. doi: 10.1002/vms3.71317 (PMC13742372; doi:10.1002/vms3.71317)
Supplement: Supplementary file 1 [file VMS3-12-e71317-s001.docx]

Case Definition, Meat Inspection, and Cyst Identification

An animal was classified as cysticercosis-positive if at least one *C. bovis* metacestode (viable, degenerated, or calcified) was identified in any inspected organ during the routine post-mortem examination. The post-mortem inspection involved visual examination, palpation, and systematic incisions into primary predilection sites, including the masseter muscles, tongue, heart, diaphragm, and active skeletal muscles, in accordance with national meat inspection guidelines. Suspected lesions were evaluated macroscopically and categorized based on their morphological appearance and viability: i. Viable (fertile) cysts: These are characterized as fluid-filled, translucent, vesicle-like structures that contain a visible white scolex. ii. Non-viable (degenerated/calcified) cysts: These are identified as opaque, yellowish, caseous, or hard calcified nodular lesions that lack a fluid-filled cavity. The viability of intact cysts was confirmed ex vivo using an evagination assay. In this process, intact, fluid-filled cysts were carefully harvested from the tissue and incubated in a 30% bovine bile-saline solution at 37°C for 1 to 2 hours to induce scolex evagination. Cysts that successfully evaginated the scolex under a stereomicroscope were confirmed as viable *C. bovis*. Species identification was based solely on gross morphological appearance and the physical dynamics of evagination. Other diagnostic methods, such as microscopic tissue squash, histopathology, or molecular testing (e.g., PCR), were not utilised in this study due to resource constraints in routine abattoir settings.
